# Irinotecan metabolite SN38 results in germ cell loss in the testis but not in the ovary of prepubertal mice

**DOI:** 10.1093/molehr/gaw051

**Published:** 2016-11-03

**Authors:** Federica Lopes, Rowena Smith, Sophie Nash, Rod T. Mitchell, Norah Spears

**Affiliations:** 1 Centre for Integrative Physiology, University of Edinburgh, EdinburghEH8 9XD, UK; 2 MRC Centre for Reproductive Health, University of Edinburgh, EdinburghEH16 4TJ, UK

**Keywords:** chemotherapy, fertility preservation, testis, spermatogonia, ovary, oocyte, gonadotoxicity, tissue culture, apoptosis, cell proliferation

## Abstract

**STUDY QUESTION:**

Does the Irinotecan metabolite 7-ethyl-10-hydroxycamptothecan (SN38) damage the gonads of male and female prepubertal mice?

**SUMMARY ANSWER:**

The Irinotecan metabolite SN38 reduces germ cell numbers within the seminiferous tubules of mouse testes at concentrations that are relevant to cancer patients, while in contrast it has little if any effect on the female germ cell population.

**WHAT IS KNOWN ALREADY:**

Little is known about the role of the chemotherapeutic agent Irinotecan on female fertility, with only one article to date reporting menopausal symptoms in perimenopausal women treated with Irinotecan, while no data are available either on adult male fertility or on the impact of Irinotecan on the subsequent fertility of prepubertal cancer patients, female or male.

**STUDY DESIGN SIZE, DURATION:**

Male and female gonads were obtained from postnatal day 5 C57BL/6 mice and exposed *in vitro* to a range of concentrations of the Irinotecan metabolite SN38: 0.002, 0.01, 0.05, 0.1 or 1 µg ml^–1^ for the testis and 0.1, 1, 2.5 or 5 µg ml^–1^ for the ovary, with treated gonads compared to control gonads not exposed to SN38. SN38 was dissolved in 0.5% dimethyl sulfoxide, with controls exposed to the same concentration of diluent. The number of testis fragments used for each analysis ranged between 3 and 9 per treatment group, while the number of ovaries used for each analysis ranged between 4 and 12 per treatment group.

**PARTICIPANTS/MATERIALS, SETTING, METHODS:**

Neonatal mouse gonads were developed *in vitro*, with tissue analysed at the end of the 4–6 day culture period, following immunofluorescence or hematoxylin and eosin staining. Statistical analyses were performed using one-way ANOVA followed by Bonferroni post-hoc test for normally distributed data and Kruskal-Wallis test followed by Dunns post-test for non-parametric data.

**MAIN RESULTS AND THE ROLE OF CHANCE:**

Abnormal testis morphology was observed when tissues were exposed to SN38, with a smaller seminiferous tubule diameter at the highest concentration of SN38 (1 µg ml^−1^, *p* < 0.001 versus control) and increased number of Sertoli cell-only tubules at the two highest concentrations of SN38 (0.1 µg ml^−1^, *p* < 0.001; 1 µg ml^−1^, *p* < 0.0001, both versus control). Within seminiferous tubules, a dose response decrease was observed in both germ cell number (mouse vasa homologue (MVH)-positive cells) and in proliferating cell number (bromodeoxyuridine (BrdU)-positive cells), with significance reached at the two highest concentrations of SN38 (0.1 µg ml^−1^, *p* < 0.01 for both; 1 µg ml^−1^, *p* < 0.001-MVH, *p* < 0.01-BrdU; all versus control). No change was seen in protein expression of the apoptotic marker cleaved caspase 3. Double immunofluorescence showed that occasional proliferating germ cells were present in treated testes, even after exposure to the highest drug concentration. When prepubertal ovaries were treated with SN38, no effect was seen on germ cell number, apoptosis or cell proliferation, even after exposure to the highest drug concentrations.

**LIMITATIONS REASONS FOR CAUTION:**

As with any study using *in vitro* experiments with an experimental animal model, caution is required when extrapolating the present findings to humans. Differences between human and mouse spermatogonial development also need to be considered when assessing the effect of chemotherapeutic exposure. However, the prepubertal testes and ovaries used in the present studies contain germ cell populations that are representative of those found in prepubertal patients, and experimental tissues were exposed to drug concentrations within the range found in patient plasma.

**WIDER IMPLICATIONS OF THE FINDINGS:**

Our findings demonstrate that the prepubertal mouse ovary is relatively insensitive to exposure to the Irinotecan metabolite SN38, while it induces a marked dose-dependent sensitivity in the testicular germ cell population. The study identifies the importance of further investigation to identify the risk of infertility in young male cancer patients treated with Irinotecan.

**LARGE SCALE DATA:**

None.

**STUDY FUNDING AND COMPETING INTEREST(S):**

Work supported by Medical Research Grant (MRC) grant G1002118 and Children with Cancer UK grant 15-198. The authors declare that there is no conflict of interest that could prejudice the impartiality of the present research.

## Introduction

Recent advances in cancer treatment have significantly increased life expectancy, especially for young patients. Since most of these oncological therapies do not have cancer cell-specific action, they can also affect healthy cells, impairing important physiological processes. One of the major concerns for younger cancer patients is the risk of infertility as a result of treatment (Anderson *et al.,* 2015). Although not every chemotherapeutic drug impairs fertility, some (e.g. alkylating agents) are recognized to be particularly gonadotoxic (Meistrich *et al.,* 1992; Meistrich, 2013). Specifically, chemotherapy drug treatment of childhood cancers can result in varying degrees of gonadotoxicity, which can negatively impact future fertility (Meirow, 2000; Chow *et al.,* 2016). Nevertheless, for many drugs, the magnitude of any potential long-term effect remains to be elucidated, for both males and females, as well as for both adult and prepubertal patients. The precise percentage of patients experiencing infertility after cancer therapy, and the degree of this dysfunction, is unknown. In the majority of cases, it is a consequence of spermatogenic impairment for men or premature ovarian failure for women. In the 0–14 years age group, cancer occurs in approximately 1 in 500 children (Cancer Research UK, 2011, www.cancerresearchuk.org, date of access 11/12/2015) and gonadotoxicity for childhood cancer survivors may only become apparent after many years, even decades, of clinical follow-up due to a failure of normal gonadal function in adulthood. The ability to identify agents and regimens that confer a significant risk of gonadal damage will enable patients and their families to make informed decisions regarding the use of available strategies for fertility preservation. Furthermore, understanding the specific mechanisms of action for the effects of different classes of chemotherapeutic drugs on the reproductive system is pivotal to the development of tailored protective tools.

Assessing fertility after chemotherapy is the first step toward any type of investigation into preserving the functionality of the reproductive system. However, in both males and females it is a difficult task that requires long-term follow-up and is complicated by the large number of co-existing variables in addition to the chemotherapy itself (i.e. type of malignancy, age, and pubertal status). To date, many clinical and experimental studies have increased our knowledge about the degree of ovotoxicity induced by several chemotherapeutic drugs (Gracia *et al.,* 2012; Levine *et al.,* 2015; Waimey *et al.,* 2015). Some studies have been able to identify the specific cellular target of each individual drug in the female gonad and the degree of ovotoxicity that results from exposure (Sanders *et al.,* 1996; Meirow, 2000; Meirow *et al.,* 2007; Ben-Aharon and Shalgi, 2012; Morgan *et al.,* 2012; Thomas-Teinturier *et al.,* 2015) . This information is of particular importance because, although prepubertal females with a good prognosis and high risk of infertility cannot opt for oocyte/embryo cryopreservation as adult women are able to do, they still have the option of ovarian cortical tissue cryopreservation in order to preserve their subsequent fertility (extensively reviewed in Oktay and Oktem, 2009; Anderson and Wallace, 2011; Wallace, 2011; Levine *et al*., 2015, Waimey *et al*., 2015). For prepubertal males, it is known that some chemotherapeutic agents impair fertility, however, in many cases, azoospermia is only a temporary outcome and after a variable length of time spermatogenesis recovers (Meistrich, 1986; Schrader *et al.,* 2001). Moreover, much of the knowledge we have about the impact of chemotherapy on spermatogenesis has been obtained from adult patients, with markedly fewer studies about the chemotherapy-induced damage to the reproductive system in male childhood cancer patients, in which spermatogenesis has not yet been established (Wallace *et al.,* 1991; Meistrich *et al*., 1992; Bordallo *et al.,* 2004; Nurmio *et al.,* 2009). While adult male patients can preserve reproductive potential using the well-established option of sperm cryobanking, for prepubertal boys the only potential option is cryopreservation of testicular tissues, a technique that is currently experimental, at the time of writing proven to work only in animal models (Picton *et al.,* 2015).

Irinotecan is a chemotherapeutic drug commonly administered to both male and female patients and represents the first and second-line therapy for the treatment of metastatic and recurrent colorectal cancer. It is also used in the treatment of several other malignancies, including lymphoma, lung, gastrointestinal and pancreatic tumours (Li *et al.,* 2014). In the late 1990s, clinical trials were set up to evaluate its use in paediatric cancers and it is now part of the treatment of refractory solid tumours in young patients (Mugishima *et al.,* 2002; Brennan *et al.,* 2014; Norris *et al.,* 2014). Irinotecan is a water-soluble synthetic version of the alkaloid camptothecin (CPT), initially isolated from the Chinese tree *Camptotheca acuminate*, and then synthesized for medical use as 7-ethyl-10-(4-[1-piperidino]-1piperidino)-carbonyl-camptothecin hydrochloride trihydrate (namely CTP-11 or irinotecan) (Mugishima *et al*., 2002). Irinotecan works as an S phase-specific inhibitor of topoisomerase I, a key nuclear enzyme for the relaxation of DNA double helix super-coiling during replication (Voigt *et al.,* 1998; Zhang *et al.,* 2004): as such, irinotecan impairs cell proliferation. *In vivo*, irinotecan is converted by hydrolysis into its active metabolite, 7-ethyl-10-hydroxycamptothecan (SN38) which is a thousand times more cytotoxic than irinotecan itself (Kawato *et al.,* 1991). Pharmacokinetic studies show plasma concentrations of SN38 in adults ranging between 0.01 and 0.1 µg ml^−1^ within the first 10 h after CPT-11 administration (Xie, 2002), while plasma concentration of SN38 documented in paediatric patients are around 0.005–0.05 µg ml^−1^ after infusion of 200 mg/m^2^ of CPT-11 per day over 3 days (Mugishima *et al*., 2002). Irinotecan results in a range of severe acute effects, such as diarrhoea, nausea, vomiting and neutropenia; however, the long-term effects of this chemotherapeutic drug metabolite are less clear.

The effect of irinotecan on fertility has not been described. One research group documented menopausal symptoms in perimenopausal women after administration of irinotecan in combination with other drugs (Tanaka *et al*., 2008). However, multiple-agent therapies render it difficult to identify the specific contribution of each individual compound, on top of which menopausal symptomatology can only be an indicative parameter of fertility. Only one study investigated the mechanism of action of irinotecan using a mouse model, showing an increase in granulosa cell (GC) apoptosis after irinotecan administration (Utsunomiya *et al.,* 2008). However these data are limited to the adult female mouse, with no information regarding the effect on earlier stages of ovarian follicles, particularly those constituting the ovarian reserve. Men undergoing chemotherapy that includes irinotecan are generically advised of a possible subsequent impairment in sperm production (Cancer Research UK, www.cancerresearchuk.org, date of access 28/2/2016), despite the fact that there are no data available regarding the role of irinotecan on spermatogenesis. Most importantly, there is no information available about future fertility following irinotecan administration to prepubertal patients of either gender.

Using an *in vitro* mouse model, we have previously demonstrated that different classes of chemotherapeutic drugs display variable degrees and mechanisms of ovotoxicity (Morgan *et al.,* 2013; Lopes *et al.,* 2014). Furthermore, since male and female germ cells have undergone markedly different early specialization supported by different populations of somatic cells, it cannot be assumed that the same drug will have the same effect on male and female gonads. Here, we have used an established mouse ovary culture model and developed an equivalent mouse testis culture model to examine the specific role of irinotecan on prepubertal male and female gonads. For this work, testicular and ovarian tissue were exposed to SN38 *in vitro*, at concentrations spanning those found in the serum of patients treated with irinotecan.

## Materials and methods

### Mice

C57BL/6 mice were kept under 14 h:10 h light:dark cycle in an approved animal facility, with food and water provided *ad libitum*. Experiments were approved by the University of Edinburgh's Local Ethical Review Committee with procedures performed in accordance with UK Home Office regulations.

### Testis culture

Testes were collected from postnatal day (pnd) 5 male mice and placed in Leibovitz (L-15, Invitrogen, UK) medium supplemented with 3 µg ml^−1^ of bovine serum albumin (BSA: Sigma-Aldrich Ltd, UK) after the removal of non-gonadal tissue. Each testis was cut into small fragments of roughly 0.5 mm^3^ using a small scalpel blade (Altomed Ltd, UK), with each piece placed on a floating polycarbonate membrane (Whatman Nucleopore Polycarbonate Membrane, Camlab Ltd, Cambridge, UK) in a 24-well plate (Grenier Bio-one, Stonehouse, UK) containing 1 ml of α-minimum essential media (MEM) culture medium (Invitrogen, UK). As with Sato and colleagues (Sato *et al.,* 2011), culture medium was supplemented with 10% knockout serum replacement (KSR, Invitrogen, UK) and incubated under a controlled atmosphere with 5% CO_2_ at 37°C for 24 h (Day 1). On Day 2 of culture, vehicle-control 0.5% dimethyl sulfoxide (DMSO; Sigma-Aldrich Ltd, UK) or SN38 (Sigma-Aldrich Ltd, UK) dissolved in DMSO, was added to the medium. Concentrations of SN38 used were: 0.002, 0.01, 0.05, 0.1 or 1 µg ml^−1^, all in 0.5% DMSO. On Day 3, testes were moved into drug-free medium. For cell proliferation experiments, culture medium was supplemented on Day 4 with 15 µg ml^−1^ of bromodeoxyuridine (BrdU; Sigma-Aldrich Ltd, UK) for the final 24 h of culture. At the end of Day 4, tissues were fixed and processed as detailed below.

### Ovary culture

Ovaries were collected from pnd 4 female mice and placed in L-15 medium supplemented with 3 µg ml^−1^ BSA for removal of non-gonadal tissue. Ovaries were placed on floating polycarbonate membranes in 24-well plates containing α-MEM culture medium supplemented with 3 µg ml^−1^ BSA and incubated under a controlled atmosphere with 5% CO_2_ at 37°C for 24 h (Day 1). On Day 2, medium was supplemented with vehicle-control 0.5% DMSO or increasing concentration of SN38: 0.1, 1, 2.5 or 5 µg ml^−1^ in 0.5% DMSO. After 24 h (Day 3), ovaries were moved into drug-free medium. For cell proliferation experiments, culture medium was supplemented with 15 µg ml^−1^ BrdU for the final 24 h of culture. Ovaries were kept in culture until Day 6, when tissues were processed for analyses as detailed below.

### Morphological evaluation

At the end of culture, testes and ovaries were fixed in Bouin‘s fluid and embedded in paraffin wax. Serial sections of 5 µm thickness were cut and stained with haematoxylin and eosin (H&E).

Three testis sections, taken from the beginning, middle and end of each piece of tissue, were photomicrographed (DMLB Leica microscope, Leica Microsystem Ltd, UK) and used for morphological examination by a blind-to-treatment assessor using ImageJ software. In each section, the total number of seminiferous tubules was noted, along with the number of seminiferous tubules that lacked visible germ cells on the basement membrane (Sertoli cell-only tubules). Within each section, the diameter of every spherical tubule was measured. Total section area and seminiferous tubule area were also recorded.

Every 6th ovarian section was photomicrographed and used for ovarian follicle counts and health assessment using ImageJ software by a blind-to-treatment assessor, as detailed in Morgan *et al*. (2013). In brief, follicles were staged as: primordial, when an oocyte with a visible germinal vesicle (GV) was surrounded only by flattened GCs; transitional, when an oocyte with a visible GV was surrounded by a mixture of flattened and cuboidal GCs; primary, when an oocyte with a visible GV was surrounded only by cuboidal GCs. Follicles were further classified as unhealthy when containing: an oocyte with eosinophilic and shrunk cytoplasm, and/or condensed nuclear chromatin; GCs, the majority of which were irregularly shaped and/or had condensed chromatin; or those follicles with a combination of unhealthy oocytes and GCs. The total number of follicles was estimated by correcting the count of any visible GV in each section using the Abercrombie formula (Abercrombie, 1946).

### Immunofluorescence

At the end of the culture period, ovarian and testis tissue was fixed in 10% neutral buffered formalin (Sigma-Aldrich Ltd, UK) and embedded in paraffin wax. Serial sections of 5 µm thickness were cut and three sections, from the beginning, middle and end of each piece of tissue, were used for immunofluorescence as previously described (Lopes *et al*., 2014). In brief, after rehydration, slides underwent antigen retrieval in sodium citrate (10 mM, pH6; Fisher Chemical, Loughborough, UK) and blocking in 20% normal goat serum in PBS (Fisher Scientific UK Ltd, UK) with 0.1% Triton X-100 (PBST) and 5% BSA. Primary antibody incubation was performed overnight at 4°C with mouse anti-mouse vasa homologue (MVH; 1:100; Abcam, UK), rabbit anti-cleaved caspase 3 (CC3; 1:500; Cell Signalling Technology, USA) or with rat anti-BrdU (1:200; Abcam, UK) either alone or alongside the antibody for MVH. Slides were incubated for 1 h at room temperature with secondary antibodies: Alexa Fluor 568 goat anti-mouse IgG1 (1:200; Invitrogen, UK) for MVH, goat anti-rabbit biotinylated (1:200; DakoCytomation, Denmark) for CC3 and rabbit anti-rat biotinylated (1:200; Vectors Lab, UK) for BrdU, with the latter two reactions followed by 30 min at room temperature with Alexa Fluor 488 streptavidin conjugate (1:200; Invitrogen, UK). Slides were counterstained with DAPI (Invitrogen, UK), mounted in Vectashield mounting medium (Vector Laboratories, USA) and photomicrographs obtained (Leica DM5500B microscope on a DFC360FX camera). Image analysis was performed with ImageJ software, with the assessor blind to treatments. The degree of expression of germ cell (MVH), apoptotic (CC3) and proliferation markers (BrdU) was given by the area of immunoreactivity relative to the section area as previously described (Lopes *et al*., 2014). Randomly selected images were used to compare manual counting of MVH positive cells versus semi-automated analysis using ImageJ software.

### Statistical analysis

Statistical analyses of data were performed using GraphPad Prism software (GraphPad Software, Inc., CA, USA). Treatments groups were checked for statistical significance compared to control. The non-parametric Kolmogorov-Smirnov test was used to assess normal distribution. For data with a normal distribution, one-way ANOVA and Bonferroni post-hoc test were applied, while data that were not normally distributed were assessed for statistical significance using the Kruskal-Wallis test followed by Dunns post-test. Pearson‘s correlation coefficient was used to determine the correlation between manual count and automated measurement of the immunofluorescence staining. Results were considered statistically significant where *p* < 0.05.

## Results

### SN38 impairs testis morphology

**Figure 1:**
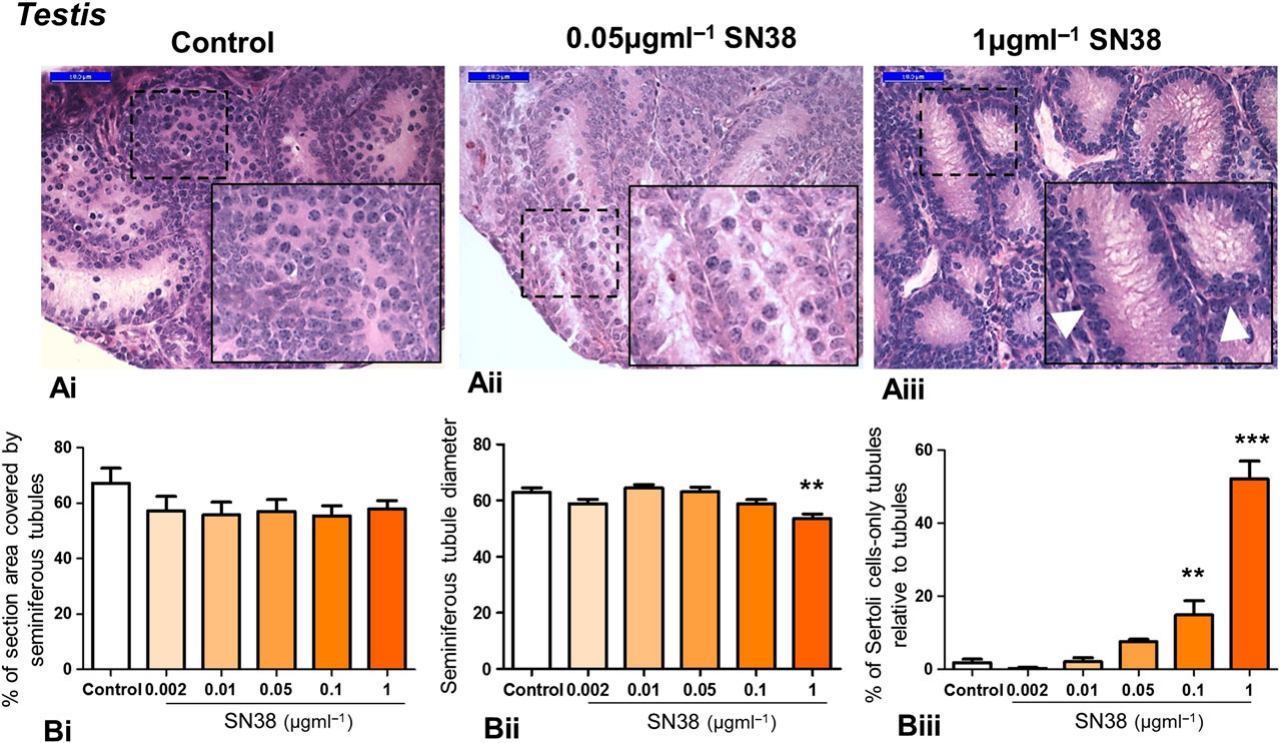
Exposure of mouse testis to SN38 affects tissue morphology. Testis tissue exposed *in vitro* to varying concentrations of the irinotecan metabolite 7-ethyl-10-hydroxycamptothecan (SN38: 0.002, 0.01, 0.05, 0.1, 1 µg ml^−1^, all in 0.5% dimethylsulphoxide (DMSO) as diluent) were processed for morphological examination. (**A**): Representative photomicrographs of haematoxylin and eosin (H&E) stained sections from 3 experimental groups: control (**Ai**), middle (**Aii**) and highest (**Aiii**) SN38 concentrations. Insets are higher magnification of framed areas. The highest SN38 concentration caused a dramatic increase in Sertoli cell-only tubules (arrowhead). (**B**): SN38 treatment did not affect the density of seminiferous tubules (**Bi**). Tubule diameter was significantly smaller in testis exposed to the highest SN38 concentration (**Bii**); and the number of Sertoli cell-only tubules increased in the two highest SN38 concentrations (**Biii**). A minimum of 3 pieces were analysed for each treatment and for the corresponding control (in 0.5% DMSO). Scale bars = 50 µm. Graphs show mean ± SEM. Data were analysed using Kruskal-Wallis test followed by Dunns post-test; ** *p* < 0.01, *** *p* < 0.001 versus control.

In order to assess whether irinotecan affects the morphology of mouse testis, tissue was treated with increasing doses of SN38 (0.002, 0.01, 0.05, 0.1 or 1 µg ml^−1^) and histological sections were assessed (Fig. 1A). Seminiferous cord structure was maintained across the experimental groups, but examination of the histological sections shows that highest concentration of SN38 led to a dramatic increase in Sertoli cell-only tubules. While the density of seminiferous tubules was unaffected by treatment (Fig. 1Bi), seminiferous tubule diameter decreased significantly at the highest SN38 concentration (Fig. 1Bii), along with a dose-response increase in the number of Sertoli cell-only tubules that reached significance at 0.1 and 1 µg ml^−1^ SN38, rising from around 2% in control to 15% and 52% of tubules respectively (Fig. 1Biii).

### Manual counting validates semi-automated measurement of immunoreactivity

**Figure 2:**
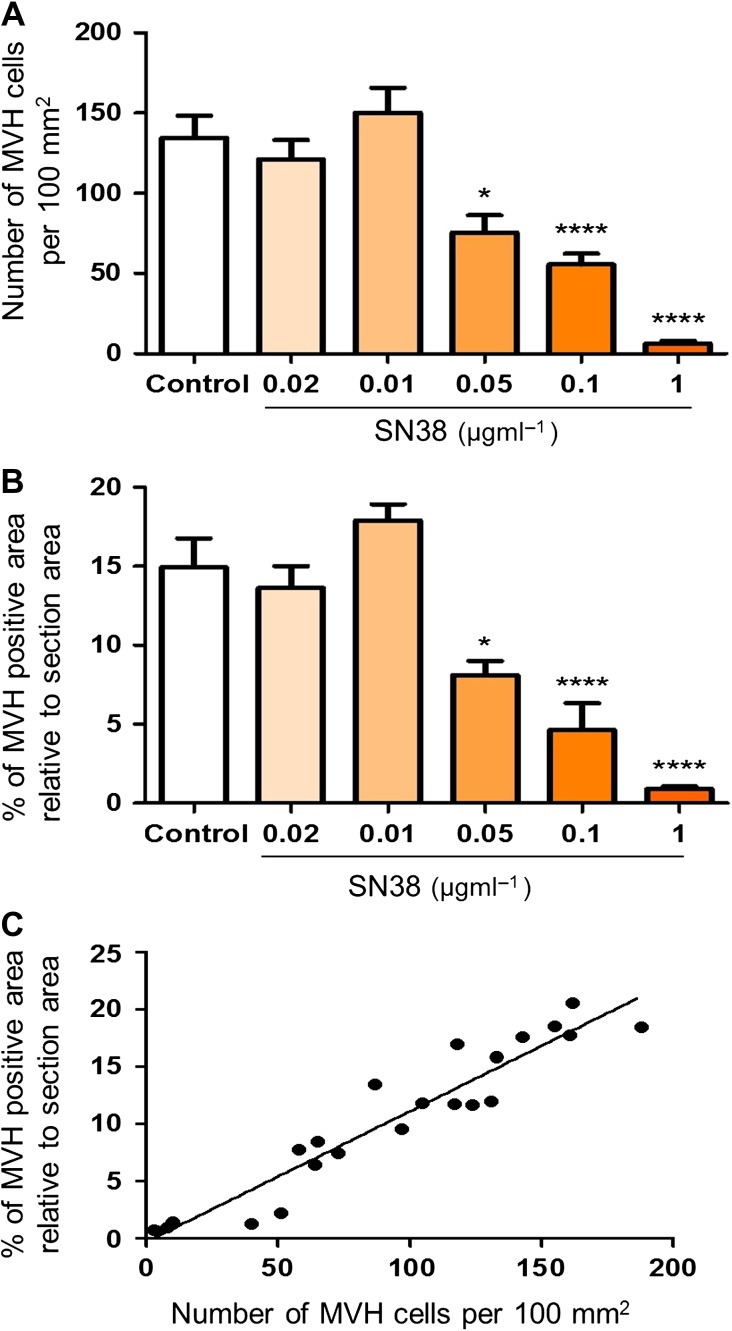
Validation of the semi-automated measurement of immunofluorescence. Immunostained sections for the germ cell marker mouse vasa homologue (MVH) were randomly selected from each experimental group and analysed using both the manual counting of germ cells and the percentage of the section area expressing the germ cell marker. A strong correlation is present between the number of germ cells per 100 mm^2^ (**A**) and the percentage of stained area (**B**) across all the experimental groups (*r* = 0.954, *p* < 0.0001) (**C**). Histograms show mean ± SEM. Data were analysed using one-way ANOVA followed by Bonferroni post-hoc test (A, B) and by Pearson's correlation coefficient (C); *p < 0.05, ****p < 0.0001 versus control.

To compare manual counting versus semi-automated measurement of immunofluorescence cells, 3–4 randomly selected testis sections immunostained for MVH for each experimental group (control, 0.002, 0.01, 0.05, 0.1 or 1 µg ml^−1^ SN38) were analysed using both systems (Fig. 2). Manual counts of MVH positive cells per section showed a significant decrease in germ cell number with a reduction from 134 cells per 100 mm^2^ in control group to 75, 55 and 6 cells per 100 mm^2^ in 0.05, 0.1 or 1 µg ml^−1^ SN38 groups, respectively (Fig. 2A). A similar decrease was demonstrated with the semi-automated measurement, where the percentage of section area expressing MVH immunostaining was reduced by 15% in control group to 8%, 4% and 0.9% in the 0.05, 0.1 or 1 µg ml^−1^ SN38 groups respectively (Fig. 2B). Both measurement systems showed that germ cell number and percentage immunostained area decreased by approximately 2, 3 and 20 fold in the groups treated with the highest SN38 concentration compared with control, demonstrating a highly significant correlation between the two methods (*r* = 0.954, *p* < 0.0001; Fig. 2C).

### SN38 affects testicular germ cell number and proliferation, but not apoptosis

**Figure 3:**
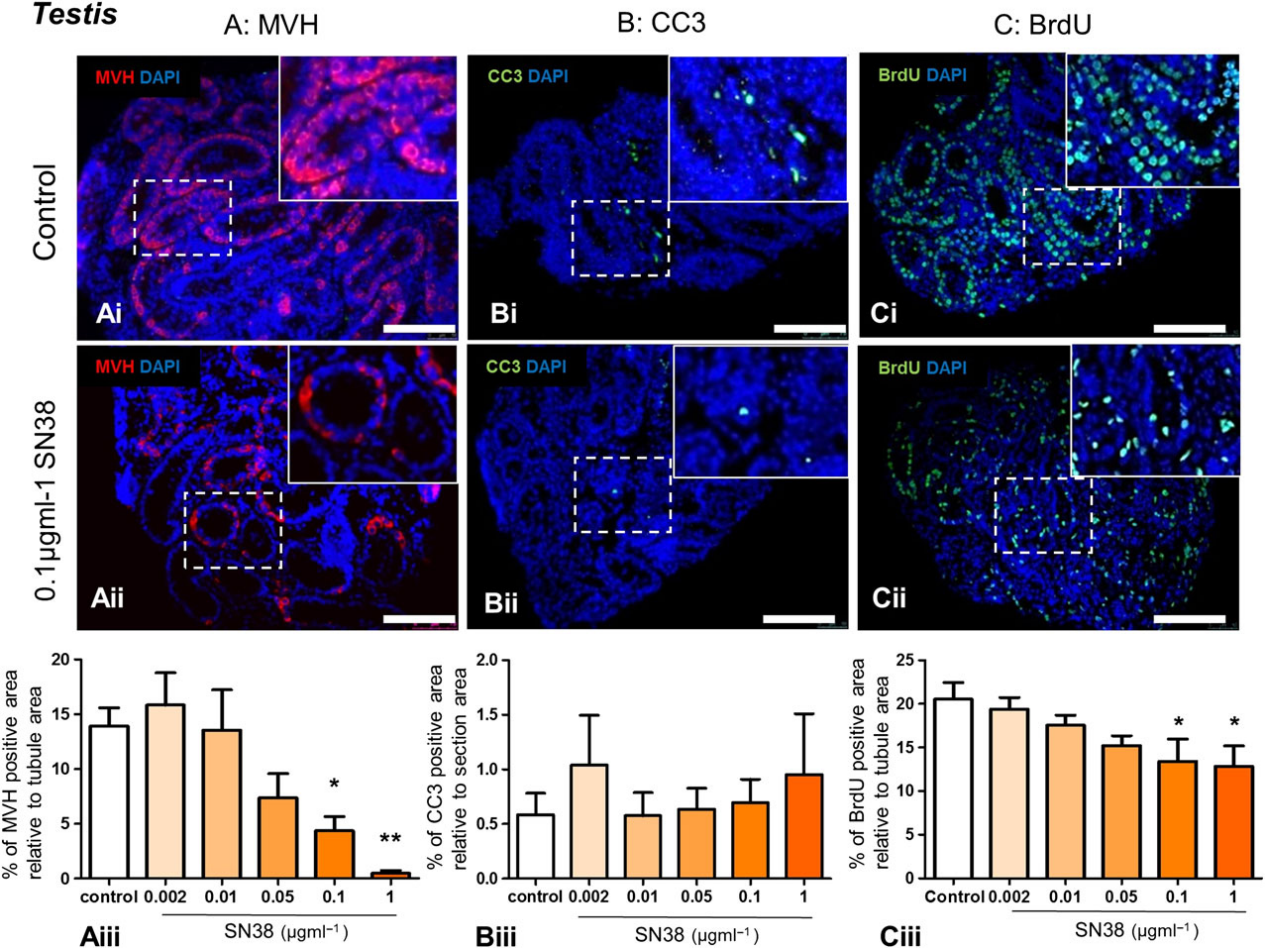
Seminiferous tubules of mouse testes treated with SN38 contain fewer germ cells and proliferating cells. Testis tissue exposed *in vitro* to varying concentrations of SN38 (0.002, 0.01, 0.05, 0.1, 1 µg ml^−1^) were analysed for the protein expression of: (**A**) germ cell marker MVH (red), (**B**) apoptotic marker cleaved Caspase 3 (CC3; green), and (**C**) proliferation marker bromodeoxyuridine (BrdU; green). Sections were counterstained with DAPI (blue). Photomicrographs of representative immunostained sections from control (**Ai**, **Bi**, **Ci**) and 0.1 µg ml^−1^ SN38 (**Aii**, **Bii**, **Cii**) groups. Insets are higher magnification of framed areas. Graphs show protein expression calculated as a percentage of tubule (**Aiii** and **Ciii**) or section (**Biii**) area. MVH and BrdU expression dramatically declined at the two highest SN38 concentrations (Aiii and Ciii), while CC3 is unaffected by drug treatment (Biii). The number of testis pieces analysed for each experimental group were between 5 and 9 in the MVH and CC3 assessments and 5–8 in the BrdU staining, all from at least 3 independent experiments. Scale bars = 100 µm. Graphs show mean ± SEM. Data were analysed using Kruskal-Wallis test followed by Dunns post-test (Aiii and Biii) and by one-way ANOVA followed by Bonferroni post-hoc test (Ciii); *p < 0.05, **p < 0.01 versus control.

Immunohistochemistry was used to assess whether SN38 affected the number of germ cells (MVH expression; Fig. 3A), the amount of apoptosis (CC3 expression; Fig. 3B) or the level of cell proliferation (BrdU expression; Fig. 3C). MVH-expression, correlating with germ cell numbers, decreased markedly in a dose-dependent manner, reaching statistical significance at concentrations of 0.1 and 1 µg ml^−1^ SN38 when compared with the control group (Fig. 3Aiii), with MVH-expression falling from 13.9% to 4.3% and 0.4% of tubule area, respectively. No difference was observed in the percentage of CC3-positive, apoptotic cells across treatments (Fig. 3Biii). There was a decrease in the percentage of proliferating cells within tubules, becoming significantly lower after exposure to 0.1 and 1 µg ml^−1^ SN38 (Fig. 3Ciii), with the BrdU expression falling from 20.5% to 13.4% and 12.8% of the tubule, respectively.

**Figure 4:**
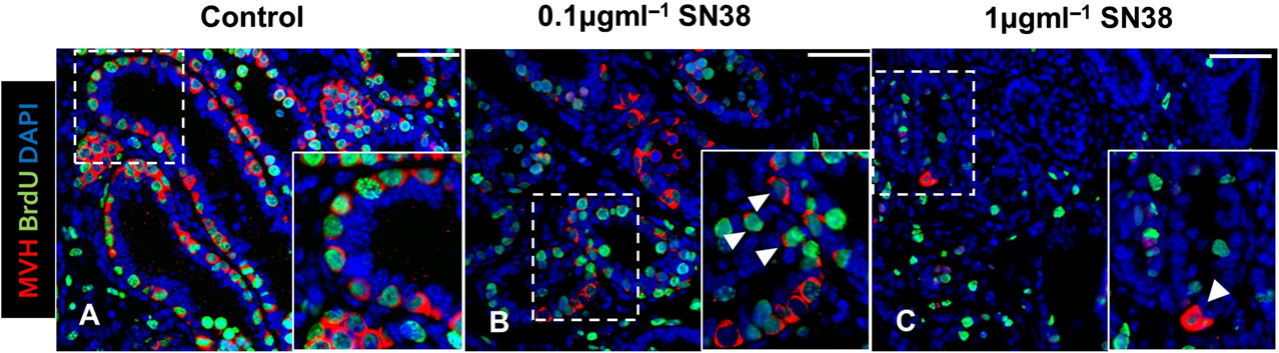
Proliferative germ cells are still present in mouse testis treated with SN38. Testis tissue exposed *in vitro* to 0.1 or 1 µg ml^−1^ SN38 were analysed for co-expression of germ cell marker MVH (red) and proliferation marker BrdU (green), with DAPI (blue) used as counterstain. Photomicrographs of representative immunostained sections from control (**A**), 0.1 µg ml^−1^ (**B**) and 1 µg ml^−1^ SN38 (**C**) groups. Insets are magnification of framed areas. The number of proliferating germ cells decreased in treated tissues, but they were still occasionally found even at in the highest SN38 concentration group (arrowheads). Scale bars = 50 µm.

Double immunofluorescence for MVH and BrdU was carried out on randomly selected sections from control tissue and from tissue exposed to the two highest concentrations of SN38, 0.1 and 1 µg ml^−1^, to determine if any of the spermatogonia remaining were still proliferating (Fig. 4). In the control group, the majority of the germ cells were actively proliferating, but, even at the highest concentration of SN38, with very few germ cells remaining, occasional proliferative germ cells could still be found (Fig. 4).

### Ovarian follicle morphology is unaffected by SN38

**Figure 5:**
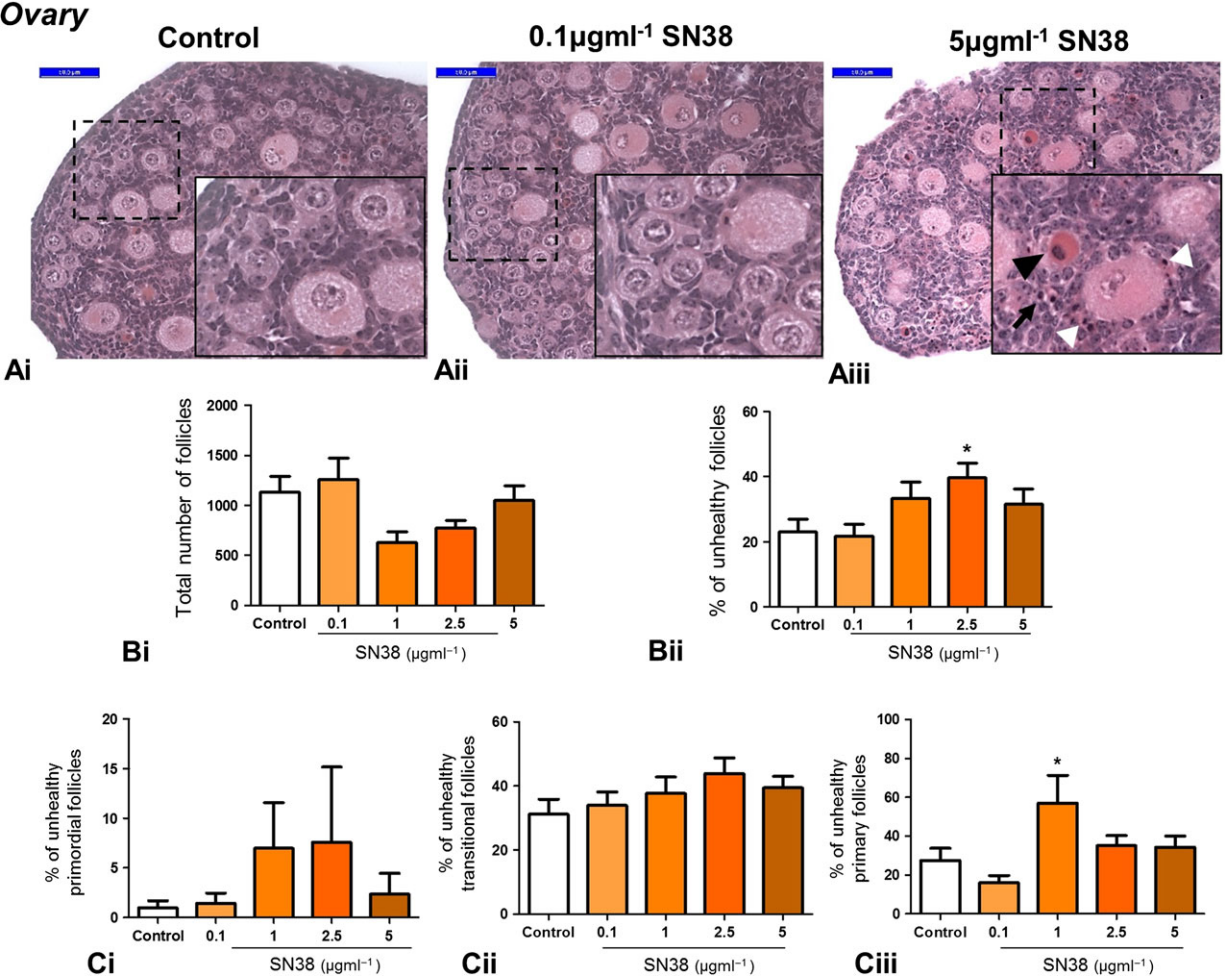
Exposure of mouse ovaries to SN38 does not impair tissue morphology. Whole ovaries exposed *in vitro* to high concentrations of SN38 (0.1, 1, 2.5, 5 µg ml^−1^) were processed for morphological examination and follicle count. Representative photomicrographs of H&E stained sections from 3 experimental groups: control (**Ai**), lowest (**Aii**) and highest (**Aiii**) SN38 concentrations. Insets are magnification of framed areas. Follicles with an unhealthy oocyte (black arrowhead) or unhealthy granulosa cells (white arrowhead), as well as degenerated stroma cells (arrow) were more often present in the highest SN38 group (Aiii). SN38 did not affect total ovarian follicle number (**Bi**) and only 2.5 µg ml^−1^ SN38 concentration increased the percentage of unhealthy follicles (**Bii**). SN38 did not affect the percentage of unhealthy primordial or transitional follicles (**Ci** and **Cii**) whilst the number of unhealthy primary follicles was significantly increased only following treatment with 1 µg ml^−1^ SN38 (**Ciii**). The number of ovaries analysed was between 8 and 12 for each treatment/control group. Graphs show mean ± SEM. Data were analysed using Kruskal-Wallis test followed by Dunns post-test (Bi and Ci) and one-way ANOVA followed by Bonferroni post-hoc test (Bii, Cii and Ciii); *p < 0.05 versus control. Scale bars = 50 µm.

To assess whether the ovarian follicle reserve was affected by exposure to SN38, neonatal mouse ovaries were cultured in the presence or absence of SN38. Initial experiments exposing ovaries to concentrations up to the highest levels found in the plasma of adult patients treated with irinotecan (0.1 μg ml^−1^) found no evidence of damage (data not shown). In order to determine if a dose-response pattern could be found, concentrations were therefore increased markedly, exposing ovaries to 0.1, 1, 2.5 or 5 µg ml^−1^ of SN38. Overall, effects of SN38 on the ovary were not observed until the concentration of SN38 was much higher than the highest concentrations found in the plasma of patients treated with irinotecan, and even then effects observed were not marked (Fig. 5). Examination of histological sections (Fig. 5Ai–iii) revealed signs of stromal cell damage only after exposure to 5 µg ml^−1^ SN38. Total follicle number was unaffected by drug treatment (Fig. 5Bi), while the percentage of follicles assessed as unhealthy was significantly increased only in the 2.5 µg ml^−1^ SN38 treatment group, rising from 23% to 40% (Fig. 5Bii). To determine whether a specific stage of early follicle development was affected by SN38 treatment, the percentage of follicles assessed as unhealthy was examined separately for primordial, transitional and primary follicles (Fig. 5C): only primary follicles exposed to 1 µm ml^−1^ SN38 showed a significant increase in the percentage deemed to be unhealthy, rising from 27% to 57% (Fig. 5Ciii).

### SN38 does not affect ovarian germ cell number, ovarian cell apoptosis or proliferation

**Figure 6:**
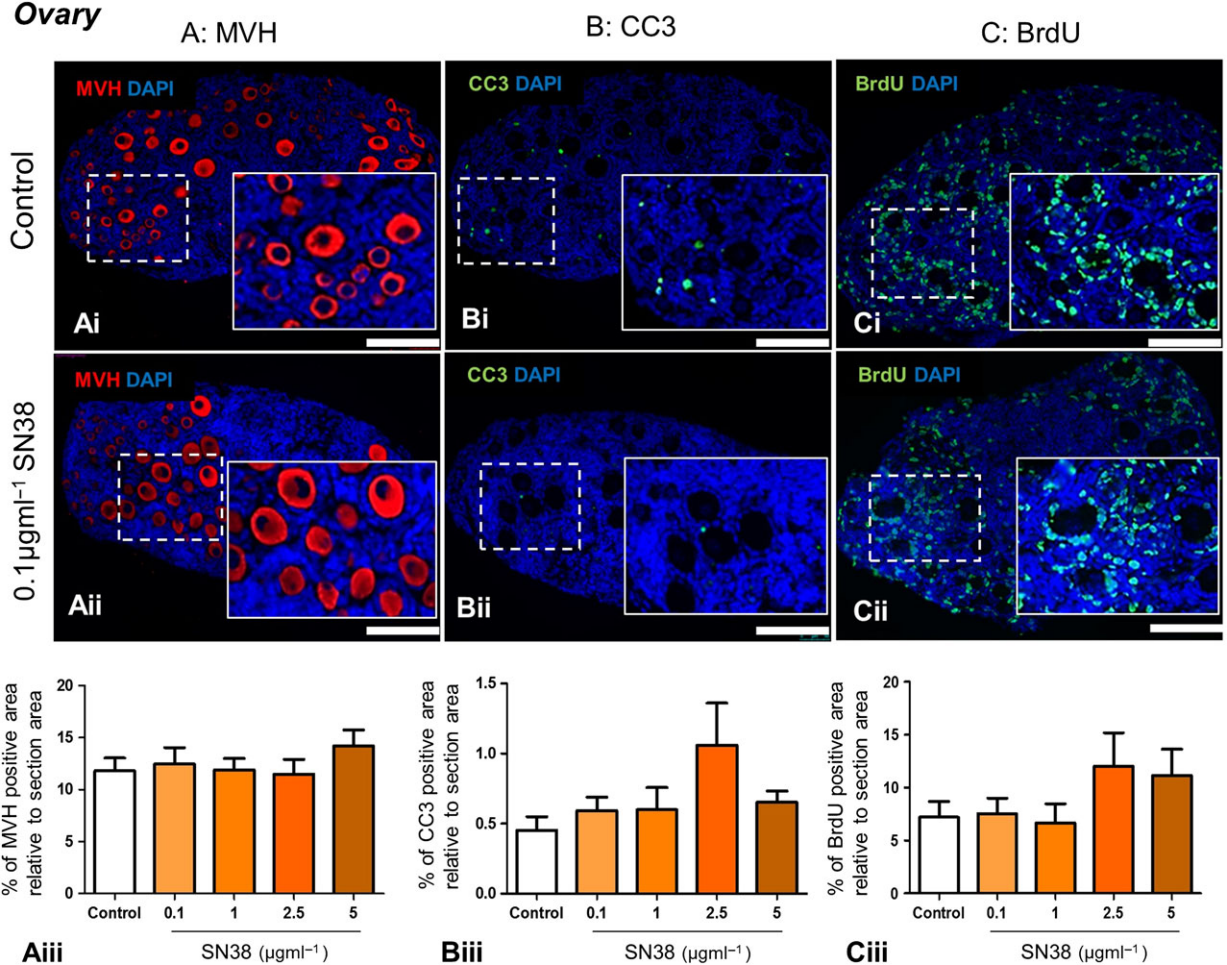
SN38 does not affect the expression of germ cell, apoptosis and proliferation markers in the mouse ovary. Whole ovaries exposed *in vitro* to high concentrations of SN38 (0.1, 1, 2.5, 5 µg ml^−1^) were analysed for the expression of: (**A**) the germ cell marker MVH, (**B**) the apoptotic marker CC3; and the (**C**) proliferative marker BrdU. Photomicrographs are representative of sections from control (**Ai**,** Bi**,** Ci**) and 0.1 µg ml^−1^ SN38 concentration (**Aii**,** Bii**,** Cii**) groups. Graphs show MVH (**Aiii**), CC3 (**Biii**) and BrdU (**Ciii**) expression levels as a percentage of section area: expression was unaffected by SN38 treatment in all cases. A minimum of 4 ovaries were analysed in each experimental group in MVH and CC3 assessments and 5 ovaries in the BrdU staining, all from at least 3 independent experiments. Scale bars = 100 µm. Graphs show mean ± SEM. Data were analysed using Kruskal-Wallis test followed by Dunns post-test.

As with the testis, the effect of SN38 exposure on germ cell number, apoptosis and proliferation was assessed using immunohistochemistry, by examining expression of the germ cell marker MVH (Fig. 6A), apoptotic cell marker CC3 (Fig. 6B) and proliferation marker BrdU (Fig. 6C). Relative to section area, the area of immunoreactivity of all three markers (MVH, CC3 and BrdU) was unaffected by the drug, even after exposure to 5 µm ml^−1^ SN38 (Fig. 6).

## Discussion

The present study shows that SN38, the active metabolite of irinotecan, is cytotoxic to male germ cells in the prepubertal mouse testis, whilst no similar effect was demonstrated in the prepubertal ovary. An *in vitro* mouse model was used to expose male and female gonadal tissue to concentrations of SN38 that are clinically relevant, covering the range of reported concentrations found in the serum of patients shortly after administration of irinotecan. Results show that the prepubertal testis is affected by exposure to the high end of clinically relevant concentrations of SN38 (0.1 µg ml^−1^ SN38 and above). In contrast, there was little effect of SN38 on the ovary even when concentrations were increased to 50-fold higher concentrations of SN38 that have been found in patients.

To the best of our knowledge, there is no published information about the effect of SN38 on the prepubertal testis or ovary. Work here shows that SN38 targeted spermatogonia, significantly and markedly reducing germ cell number, leading to a 3.5-fold and 35-fold reduction in MVH protein expression after exposure to 0.1 and 1 µg ml^−1^ SN38 respectively, accompanied by an equally dramatic 7.5- and 26-fold increase in Sertoli cell-only tubules. SN38 is a potent inhibitor of cell proliferation (Bomgaars *et al.,* 2006). It is believed that the high growth rate of the germ cell population renders the testis particularly sensitive to chemotherapeutic drugs whose principal mechanism of action is to impair the replication ability of cancer cells. Here, germ cells decreased significantly in tubules of testis tissue exposed to high SN38 concentrations, as did all proliferating cells in the seminiferous tubule. However, the high proliferation rate may not be the only explanation for the specific vulnerability of spermatogonial germ cells, since other factors could also be involved. This could include factors intrinsic to the germ cells or indirect actions via somatic cells that impair signalling to germ cells, resulting in germ cell loss. Indirect effects on spermatogonial proliferation and differentiation via the somatic Sertoli cells have been shown (Brilhante *et al.,* 2012). For example, KIT-ligand (also known as stem cell factor) produced by Sertoli cells is required to support differentiation of spermatogonia through interaction with the c-kit receptor on differentiated spermatogonia (Ohta *et al.,* 2000). In addition, irradiation in the rat testis has been demonstrated to block spermatogonial differentiation as a result of damage to the somatic compartment (Zhang *et al.,* 2007). Results here suggest that chemotherapeutic drugs have differential effects on germ cells rather than Sertoli cells in terms of cell loss within the seminiferous tubules; however, effects of SN38 exposure on other testis somatic cell types (e.g. Leydig or peritubular myoid cells) require further investigation.

Whilst the present study has demonstrated clear and dramatic effects of SN38 on the germ cells of the prepubertal mouse testis, extrapolation of these results to prepubertal human must take into account variations in spermatogonial development between these species. The germ cell population in rodents arises from spermatogonial stem cells (SSCs) that are generally classified as A_single_, which regularly, although infrequently, self-renew and A_paired_/A_aligned_ which are committed progenitor cells with only a few cycles of self-renewing divisions (Hermann *et al.,* 2010). In primates, including humans, two separate spermatogonial cell populations make up the SSC pool: a stem cell reserve with a low/none proliferative activity under physiological conditions (A_dark_) and a separate functional pool of highly proliferative progenitors (A_pale_) (Mitchell *et al.,* 2009). However, functional and molecular similarities and/or differences between human and rodent SSCs are still to be fully elucidated, mainly because of the lack of information about the regulation of human SSCs (Nagano *et al.,* 2002; Wu *et al.,* 2009). As such, species-specific differences in sensitivity to cytotoxic agents would require further investigation, possibly by using an experimental animal species more closely related to humans. As with all *in vitro* work, it will be important to perform *in vivo* studies to further validate our findings. These results provide valuable information about specific effects of exposures, based on dosage and timing, able to inform any future *in vivo* studies.

In addition, the present model investigates the effect of SN38 at 4–6 days after a single exposure, as opposed to administration over several cycles, as in the regimens used in patients. In this respect, the present results may underestimate the fertility impairment that may occur after repeated Irinotecan administration to young male cancer patients (Ise *et al.,* 1986; Nurmio *et al*., 2009). Importantly, amongst the very few remaining spermatogonial cells exposed to high SN38 concentrations, some retained their proliferative ability, leaving open the possibility that the seminiferous tubules could be repopulated over time, giving the potential for future fertility.

It has been proposed that male gonads are more sensitive to chemotherapy than female gonads (Ajala *et al.,* 2010). Work here has also evaluated the impact of SN38 on the ovarian follicle reserve and on early stages of follicle development. Damage to these quiescent/early growing ovarian follicles can have a major impact on the subsequent reproductive capability of females, particularly on long-term fertility. However, data here suggest that irinotecan administration is unlikely to impact on female fertility. No sign of damage was observed when mouse ovarian tissue was exposed to concentrations of SN38 comparable to those found in cancer patients. Even when SN38 concentrations were further increased to up to 50-fold higher concentrations than those found in patient plasma, there was little effect, with no morphological changes evident in primordial or early growing follicles, and with apoptosis and cell proliferation unaffected by drug exposure. Utsunomiya *et al*. (2008) also failed to find damage in small or medium follicles in response to irinotecan administration in mice, although apoptosis of GCs was observed in larger follicles. Here, it is impossible to exclude the possibility that SN38 has produced subtle damage to early-stage follicles, the effects of which might not become apparent until later developmental stages, but the simplest and most parsimonious hypothesis is that early ovarian follicle stages are not sensitive to SN38 insult.

Work here used a new tissue culture system to support short-term survival and development of the early neonatal testis *in vitro*, modified from ovary culture systems used by our laboratories (Morgan *et al*., 2013; Lopes *et al*., 2014) and other testis culture systems (Sato *et al*., 2011). Examination of the control photomicrographs and data show that the culture system maintains the structural integrity of the seminiferous tubules, and that the tubules contain proliferating germ and somatic cells.

In summary, we have investigated the gonadotoxicity of irinotecan, a chemotherapeutic drug widely administered to patients of both sexes and of all ages, prepubertal and adult. Results show that the irinotecan-metabolite SN38 results in damage to testes, significantly affecting germ cells following exposure to clinically relevant concentrations of SN38, in contrast to only minor effects on the ovary, with no effects on germ cell number even following exposure to 50 times higher concentrations of SN38 than those reported to date in patients following irinotecan administration. As such, our results using a prepubertal mouse model could indicate that germ cells in the ovaries of prepubertal girls may be less susceptible to damage by irinotecan administration than those in the testes of prepubertal boys; however, further studies, using non-human primate or human models, are necessary to confirm these results. In addition, an examination of long-term effects of irinotecan/SN38, and investigation into ways of protecting against damage, is of major importance. The retention of some proliferative germ cells after exposure to SN38 does allow for the possibility of germ cells repopulating the seminiferous tubules, which could in turn lead to recovery of fertility, although this will require further study. More generally, our results highlight the fact that chemotherapy drugs can have differential effects on the gonads of males and females.
